# Diatoms and Their Microbiomes in Complex and Changing Polar Oceans

**DOI:** 10.3389/fmicb.2022.786764

**Published:** 2022-03-25

**Authors:** Reuben Gilbertson, Emma Langan, Thomas Mock

**Affiliations:** ^1^School of Environmental Sciences, University of East Anglia, Norwich Research Park, Norwich, United Kingdom; ^2^The Earlham Institute, Norwich Research Park, Norwich, United Kingdom

**Keywords:** diatoms, psychrophile, genomics, meta-omics, polar winter, climate change, adaptive evolution, microbiomes

## Abstract

Diatoms, a key group of polar marine microbes, support highly productive ocean ecosystems. Like all life on earth, diatoms do not live in isolation, and they are therefore under constant biotic and abiotic pressures which directly influence their evolution through natural selection. Despite their importance in polar ecosystems, polar diatoms are understudied compared to temperate species. The observed rapid change in the polar climate, especially warming, has created increased research interest to discover the underlying causes and potential consequences on single species to entire ecosystems. Next-Generation Sequencing (NGS) technologies have greatly expanded our knowledge by revealing the molecular underpinnings of physiological adaptations to polar environmental conditions. Their genomes, transcriptomes, and proteomes together with the first eukaryotic meta-omics data of surface ocean polar microbiomes reflect the environmental pressures through adaptive responses such as the expansion of protein families over time as a consequence of selection. Polar regions and their microbiomes are inherently connected to climate cycles and their feedback loops. An integrated understanding built on “omics” resources centered around diatoms as key primary producers will enable us to reveal unifying concepts of microbial co-evolution and adaptation in polar oceans. This knowledge, which aims to relate past environmental changes to specific adaptations, will be required to improve climate prediction models for polar ecosystems because it provides a unifying framework of how interacting and co-evolving biological communities might respond to future environmental change.

## Introduction

Polar oceans are major drivers of the global carbon pump, circulation of nutrients and reflection of solar radiation (Murata and Takizawa, 2003; Sigman et al., 2010; MacGilchrist et al., 2014; Laufkötter et al., 2018; Wadham et al., 2019). Despite the global importance of both the geophysical and biological aspects of the polar oceans, they are critically understudied. In addition to consistently low temperatures, polar oceans comprise a multitude of stressors such as extremes in solar irradiance, salinity and UV radiation (Boyd, 2002). While the Arctic and Antarctic have similar climates due to the high latitudes, each ecosystem is characterized by unique geography which shapes the local environment. The Arctic Ocean is surrounded by large shelf areas connected to land masses and annually undergoes dramatic changes in the extent of sea ice. Conversely, the Southern Ocean is a merry-go-round system characterized by strong latitudinal gradients of temperature (Oceanic fronts) isolating the Antarctic continent (Hansom and Gordon, 1998; Maksym, 2019). These fronts have recently been discovered to be responsible for ecotypic differentiation and speciation in the endemic and pelagic Southern Ocean diatom *Fragilariopsis kerguelensis* (Postel et al., 2020). Hence, they contribute to creating and maintaining diatom biodiversity in the Southern Ocean. Wherever there are fewer barriers such as in the Arctic Ocean, there is likely more exchange (e.g., gene flow) between microbial populations. Their terrestrial landscapes also differ. For example, higher vascular plants have colonized around 75% of the Arctic (Walker et al., 2005; Iversen et al., 2015), compared to a limited number of plant species across the Antarctic (Pointing et al., 2015; Singh et al., 2018). Despite these differences, most of the primary productivity and nutrient cycling for both ecosystems takes place in marine microbial communities inhabiting their associated oceans (Nelson et al., 1987; Smythe-Wright et al., 2010).

Polar microbiomes, mainly composed of diverse microalgae and their associated prokaryotes, are of particular importance in polar primary productivity and nutrient cycling and are the base of the highly productive polar food webs (Stretch et al., 1988; Harrison and Cota, 1991; Boyd, 2002; Bluhm and Gradinger, 2008; Aslam et al., 2012; Petrou et al., 2014; Hayward and Grigor, 2020). Diatoms are the most abundant and diverse group of eukaryotic polar phytoplankton and are key components of both pelagic and sea-ice habitats (Thomas and Dieckmann, 2002; Kooistra et al., 2007; Armbrust, 2009; Bracher et al., 2009; Tréguer et al., 2018; Trefault et al., 2021), as they thrive in seasonally mixed, cold and nutrient-rich water, characteristic of the polar oceans. Thus, polar oceans are their preferred environment where they outcompete many other phytoplankton groups, at least under past and current climatic conditions. Reasons for the dominance of diatoms in polar oceans include: (a) the ability to “boom and bust”, which matches the response required to thrive under extreme seasonality, (b) an increased abundance of genetic elements that enhance an adaptive response (i.e., transposable elements), (c) differential allelic expression and (d) multiple sources of genes from endosymbiotic (EGT) and horizontal gene transfer (HGT), both of which contribute to metabolic plasticity and therefore facilitate specific adaptations (Martin et al., 1998; Sjöqvist and Kremp, 2016; Mock et al., 2017). Given their prevalence in polar phytoplankton microbiomes, initial research focused on understanding their physiological mechanisms for survival under extreme conditions. The advent of genomics tools and methods has influenced later research with a focus on identifying the underpinning genes and pathways (Sackett et al., 2013; Palenik, 2015).

Molecular dating of the emergence of adaptations and radiations in polar species coincide with major geological events, such as the opening of the Drake Passage (∼35 Mya), which resulted in the Antarctic Circumpolar Current, and subsequent isolation and freezing of Antarctica (Suto et al., 2012; Benoiston et al., 2017). Generally, between the Eocene and Oligocene (∼34 Ma), the Earth cooled, and changes in upwelling patterns created favorable conditions for diatoms, especially in the Southern Ocean, where the evidence of the rise and sustained dominance of diatoms in the water column is found in large siliceous ooze deposits in the area (Salamy and Zachos, 1999; Dutkiewicz et al., 2015; Benoiston et al., 2017). All microbial life was affected by this global cooling, including the divergence of polar clades of *Chlamydomonas sp.* from temperate lineages (Zhang et al., 2020), the Atlantic *Chaetoceros* Explosion (ACE), and global radiation in soil diatoms (Suto et al., 2012; Pinseel et al., 2020). It has been suggested that the rise in diatom abundance and subsequently primary production, specifically *Chaetoceros*, during this time enabled organisms in higher trophic levels such as zooplankton and marine mammals to thrive and diversify (Suto et al., 2012). These data highlight that major changes in marine microbial communities are inextricably linked to climate cycles and can have far-reaching implications on the rest of the food chain (Falkowski, 1998; Suto et al., 2012). Just as diversification events such as the ACE can be observed in fossil records, the genetic code of marine organisms can show how they adapted and evolved to survive changing climates. Understanding the evolution of diverse polar diatom genomes and their associated microbes can therefore provide insight into past and current climate conditions, potentially improving models based on either traits or functional types, especially due to the rapid nature of climate change in polar ecosystems (Kwok, 2018). It appears that warming polar oceans have so far been generally beneficial for polar phytoplankton populations as consistently increasing temperatures have extended the season in which polar phytoplankton can grow but have so far been small enough to prevent significant encroachment of invasive species (Arrigo et al., 2008; Erwin, 2009). Increasing temperatures are generally resulting in prolonged stratification and growing seasons, ocean acidification and reduced upwelling creating conditions which some phytoplankton are unable to adapt to, opening niches for invasive species (Vincent, 2010; Boyd et al., 2016). However, the polar oceans are continuing to warm and the wider effect this will have on diverse polar microbiomes in the future is not well known. Unlike diatom fossil records there are no historical databases built on omics data, without a reference of the current diversity of polar microbiomes and associated functional traits, our ability to predict changes is limited. To combat this, we suggest an increase in metaomics sequencing of environmental communities from the polar regions to provide a background of current populations to relate future changes to.

However, evolutionary genomics with polar microbes is still in its infancy but will be necessary to improve predictions of key species’ responses to climate change (Waldvogel et al., 2020) which could have significant effects on the food-web structure and biogeochemical cycles of elements, as seen during past major geological events (Suto et al., 2012). Even intraspecific changes in biodiversity will have knock-on effects on the carbon cycle, as the changing polar ocean likely selects for different strains (e.g., ecotypes) with different traits impacting food web dynamics and the cycling of elements especially if keystone groups such as diatoms are affected (Field et al., 1998; Wolf et al., 2019). Thus, forecasts for how this rapid climate change will impact polar ecosystems remain incomplete unless we improve our understanding of how the polar environment has shaped the evolution and biodiversity of polar organismal communities (Bindoff et al., 2007; Steig et al., 2009; Lee, 2014; Mock et al., 2016; Murphy et al., 2016; Verde et al., 2016; Brown et al., 2019).

Consequently, to address the uncertainty of the effect future climate change will have on biodiversity change and loss in polar marine ecosystems, integrative approaches are required. We think they should be based on sequencing data because they provide comprehensive insights into microbial functional diversity and how this diversity might change due to selection driven by climate change. However, our current molecular knowledge about polar microbes is very limited because of (a) lack of diverse polar model species for cell biology and therefore fundamental insights into their biochemical adaptation and molecular evolution, and (b) a very limited number of environmental sequencing initiatives to reveal genetic and genomic biodiversity of polar microbes. The aim of this review paper, therefore, is to reflect on what we have learned so far from the omics resources currently available from limited model psychrophilic microalgae, specifically *Fragilariopsis cylindrus*, and their associated microbiomes in the context of future advances exemplified by developments in neighboring fields such as plant sciences, microbiome research, and macroecology. In addition to highlighting some of the latest advances in polar diatom molecular biology, we provide suggestions as to how to build bridges to neighboring disciplines to fill gaps in our knowledge and therefore to advance our field for tackling the challenges mentioned above.

### Using *Fragilariopsis cylindrus* as a Model to Reveal How Marine Phytoplankton Are Adapted to the Polar Climate

Although diatoms are the most species-rich group of algae, diatom research has immensely benefited from model species such as *Thalassiosira pseudonana* and *Phaeodactylum tricornutum* (Falciatore et al., 2020). However, due to limitations given by the nature of both models (e.g., small and streamlined genomes, limited evidence for sexual reproduction, mesophiles, in culture for many decades), additional diatom species have been developed into models. One of them is *F. cylindrus*, which is the first and only eukaryotic psychrophile so far that is genetically tractable (Faktorová et al., 2020) with a sequenced genome, and grows well under laboratory conditions (Mock et al., 2017). Hence, it has been used to study physiological and molecular adaptation to polar conditions. Significant seasonality in light and the overall low temperatures are amongst the strongest selecting agents shaping the evolution and adaptation of *F. cylindrus* and likely other polar diatoms. In this section of the review paper, we will focus on molecular data gained from the sequencing of polar diatoms, mainly *F. cylindrus*, and how this molecular data compares to that from other psychrophilic microbes, and mesophilic model diatom species.

### The Dark Polar Winter

**FIGURE 1:**
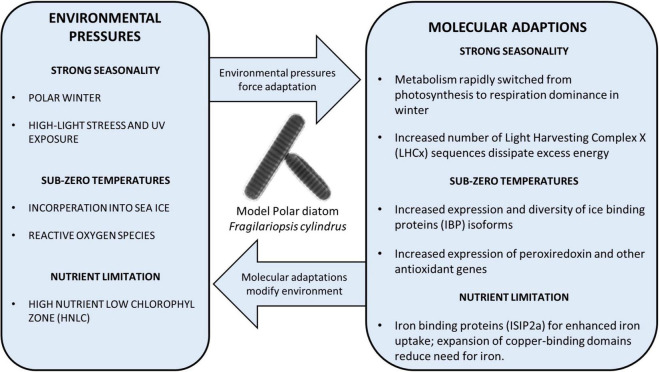
Diagram showing examples of the adaptations of polar diatoms to a variety of polar environmental stressors, discovered using the model diatom *F. cylindrus*. Directional arrows display the pressure from the environment and the feedback/modification of the environment by polar diatoms.

In most temperate and polar aquatic environments, especially coastal waters, diatoms are typically one of the first taxa groups to bloom with the changing seasons by taking advantage of renewed nutrients and increased photosynthetically active radiation (PAR) (Soreide et al., 2010; Kvernvik et al., 2018). Polar diatoms are no exception, due to mechanisms allowing them to survive the polar winter with the necessary cellular components ready and waiting for the return of spring (Peters and Thomas, 1996; van de Poll et al., 2020). However, the molecular mechanisms underpinning these adaptations have been a mystery. Polar phytoplankton have been documented to employ a diverse set of strategies, such as forming resting spores, heterotrophy and respiration of stored lipids (Bunt and Lee, 1972; Reeves et al., 2011; McMinn and Martin, 2013; Schaub et al., 2017). The traditional view of a pause in biological activity has recently been challenged as more data during the winter months is collected, indicating that there is stable biological activity (Berge et al., 2015). This is suggesting a diverse polar microbial community with multiple mechanisms of survival in prolonged darkness. The addition of detailed transcriptomics and proteomics data from *F. cylindrus* cultured in complete darkness has enabled researchers to explore genetic variation, and how it could explain the physiological activity in prolonged darkness. Within 24h of the onset of darkness, there was a significant reduction in the abundance of light-harvesting protein complexes (LHCs), which transfer light energy to the photosystem reaction center (Kennedy et al., 2019). Despite the sudden widespread downregulation in the abundance of LHCs, these complexes are maintained at a low basal level in the absence of light. This coincided with the upregulation of proteins affiliated with glycolysis, the TCA cycle and the Entner–Doudoroff pathway, suggesting a coordinated shift from photosynthesis to cellular respiration to maintain essential functions for survival (Figure 1; Kennedy et al., 2019). This large response in the proteome corroborates data on differential expression in the *F. cylindrus* transcriptome in response to prolonged darkness (Mock et al., 2017). Dynamic regulation of the proteome and transcriptome to significant changes in light intensity does not appear to be exclusive to *F. cylindrus*. For example, the model psychrophile Chlamydomonas raudensis and the natural community around it, also maintained photosystem complexes at a basal level when cultured in dialysis bags in Lake Bonney (McMurdo Dry Valleys, Antarctica) during the transition to polar night conditions (Morgan-Kiss et al., 2016). Despite this study focusing on a different ecosystem and a distantly related species from diatoms, the same molecular response was discovered, suggesting co-adaptation to the polar climate across distinct phytoplankton communities.

After months of darkness, the sea-ice thickness begins to decline in the spring and more light can reach diatom populations in the surface ocean eventually becoming sustained high light during the summer months. The *F. cylindrus* genome encodes significantly more LHCx genes, a subset of LHC sequences that are induced by high-light stress, than the model temperate diatoms *T. pseudonana* and *P. tricornutum*. The evolutionary expansion and upregulation of LHCx genes, known to reduce high-light stress, suggests their key role in the adaptive evolution to the polar light climate (Figure 1; Mock et al., 2017). Interestingly, the LHCx gene family is also expanded in the mesophilic alga *Aureococcus anophagefferens*, enabling this species to rapidly form blooms in high-light coastal environments (Gobler et al., 2011; Mock et al., 2017). Thus, to be prepared for high-light environments after a period of darkness, polar diatoms appear to be downregulating a subset of photosynthesis-associated genes accompanied by increasing cellular respiration. This combination of acclamatory metabolic processes enables them to survive the polar winter in an active state (Berge et al., 2015) and take full advantage of the spring and summer high-light conditions, especially in open water by upregulation of LHCx genes. These results show the usefulness of genomic data in researching how phytoplankton adapted to the physical polar environment, and the extent to which adaptations are possibly unique to specific taxa groups, giving more insight into the potential impact climate change will have on diverse polar phytoplankton communities.

### Freezing

#### Ice-Binding Proteins

Ice-binding proteins (IBPs) are molecules that act on the interface between ice and water (Dolev et al., 2016). Their activity can reduce the freezing point and inhibit ice-crystal growth altogether. IBPs have been found in a diverse array of taxa across the world from insects to fish, with the majority found in species living in Arctic and Antarctic ecosystems such as oceans, frozen lakes and glaciers (DeVries and Wohlschlag, 1969; Raymond, 2014; Jung et al., 2016; Vance et al., 2019). The structure of IBPs is diverse throughout the domains of life, but the general function is conserved, suggesting multiple independent origins (Dolev et al., 2016).

Many diatom species are incorporated into the sea ice each year (Eicken, 1992; Thomas and Dieckmann, 2002). When frozen into forming sea ice, diatoms are enclosed into interconnected channels and pockets filled with brine of high salinity. To maintain an aqueous habitat inside sea ice, polar diatoms are known to produce IBPs and extracellular polymeric substances (EPS) to influence the physical state of their surrounding icy environment (Hoagland et al., 1993; Bayer-Giraldi et al., 2010; Lyon and Mock, 2014; Raymond, 2014; Arrigo et al., 2017; Aslam et al., 2018; Liang et al., 2019; Vance et al., 2019). There is evidence that the IBPs they secrete help maintain the aqueous state of the brine channel system to ensure access to nutrients from the seawater underneath through diffusive and convective transport processes (Raymond et al., 2009; Dolev et al., 2016). One interesting aspect of the functional conservation of IBPs is the Domain of Unknown Function 3494 (DUF3494), which has been identified in a large range of taxa in a variety of cold habitats. Despite significant sequence divergence, all homologs share the biophysical ability to reduce the growth of ice crystals (Vance et al., 2019; Raymond et al., 2021). Phylogenetic analysis of IBP sequences does not correlate with 18S-based phylogeny, which suggests that horizontal gene transfer (HGT) may have been a vector for the DUF3494’s widespread presence (Keeling and Palmer, 2008; Bayer-Giraldi et al., 2010; Raymond and Kim, 2012; Mock et al., 2017; Raymond and Morgan-Kiss, 2017; Vance et al., 2019). HGT in sea-ice communities is considered to be facilitated by the proximity of organisms in the narrow brine-channel system in combination with a strong selection pressure imposed by the harsh environmental conditions such as high salinities and subfreezing temperatures (Raymond et al., 2009; Raymond and Kim, 2012). Their important role in the adaptive evolution to the sea-ice habitat is supported by the fact that they usually expand (e.g., gene duplications) once they have been acquired *via* HGT (Mock et al., 2017; Raymond and Morgan-Kiss, 2017). Hence, many polar organisms have more than a single IBP gene encoded in their genomes such as *F. cylindrus* and *Chlamydomonas sp*. ICE-L.

The genome of *F. cylindrus* encodes 11 unique IBP isoforms, several of which are significantly upregulated under freezing temperatures and elevated salinity (Figure 1; Mock et al., 2017). Isoform 11 from *F. cylindrus* (F*c*IBP11), despite having only moderate activity, can bind to multiple planes of ice crystals (Kondo et al., 2018). In addition to *Fc*IBPs containing the DUF3494, predicted to inhibit ice crystallization, several proteins such as *Fc*IBP-1, have transmembrane domains and are therefore hypothesized to protect the cell membrane from ice crystals (Mock et al., 2017). However, alternative roles are equally likely, such as sensing the formation of ice crystals and therefore initiating the appropriate physiological response to mitigate the impact of ice-crystal formation on the integrity of cellular structures.

**FIGURE 2:**
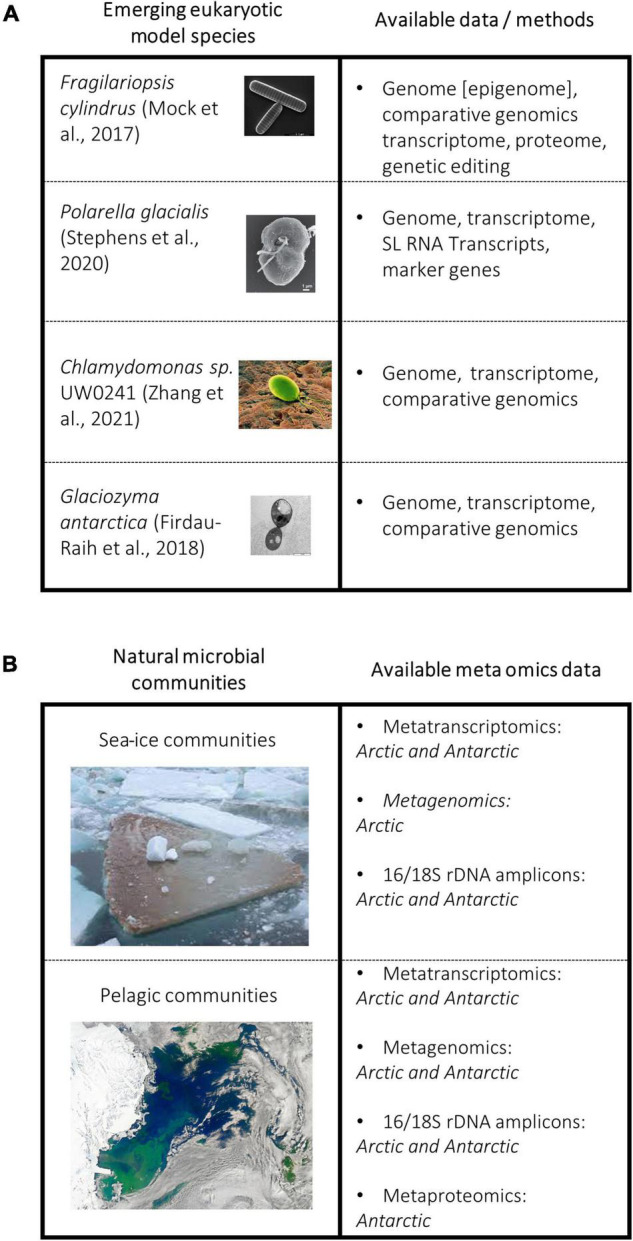
**(A)** Non-comprehensive figure showing selected model species of core polar taxa groups and a summary of available data/methods for each model system. **(B)** Polar ecosystems and available meta-omics datasets for each.

Encoding multiple isoforms of antifreeze proteins to a variety of ice planes is evident in other polar eukaryotes, such as the psychrophilic yeast *Glaciozyma antarctica* (Figure 2) and the green algae *Coccomyxa subellipsoidea* (Figure 2; Blanc et al., 2012; Firdaus-Raih et al., 2018). The genome of the psychrophilic green alga *Chlamydomonas sp.* ICE-L, the first polar green algae sequenced, also contains expanded IBP gene families. All 12 IBPs in the genome of *Chlamydomonas sp.* contain the DUF3494, and again these sequences show a closer phylogenetic relationship to that of bacteria (Zhang et al., 2020). Polar diatoms and the wider microbiome produce EPS to work in synergy with IBPs by creating disorder in ice-crystal formation leading to retention of salinity, effectively reducing the freezing point (Ewert and Deming, 2011; Krembs et al., 2011; Mykytczuk et al., 2013; Aslam et al., 2018; Raymond-Bouchard et al., 2018; Torstensson et al., 2019). By having multiple IBP isoforms with a diverse range of functions and applications to different conditions, the cell appears to be able to sense the icy environment when it forms, which likely induces diverse protection mechanisms in which IBPs play a key role.

#### Radical Oxygen Species and the Role of Trace Metals

Low temperatures generally reduce enzyme kinetics. Because of slow kinetics, excess free radicals accumulate within the cell causing dangerous levels of oxidative stress which can seriously damage cellular components, including DNA and membranes (Wingsle et al., 1999). As mentioned above, *F. cylindrus* displays a divergent expression of homologous alleles under varying environmental conditions. The most divergently expressed allele set across all tested conditions, including freezing temperatures, is glutathyamine s-transferase I (GST-1), a scavenger for free radical oxygen species (ROS). Thus, this enzyme is a good example that diverged expression of alleles might be driven by environmental pressures (Mock et al., 2017). Similarly, *F. kerguelensis* overexpressed transcripts for peroxiredoxin (PrxQ) to inhibit a diverse set of peroxides (Moreno et al., 2020). Similar responses across other psychrophiles include growth changes, upregulating expression of other antioxidant proteins such as specific catalases, overexpression of antifreeze proteins, and superoxide dismutases (Wong et al., 2019; Zhang et al., 2020). The latter converts superoxide radicals to more stable species (Miteva-Staleva et al., 2011; Scholz et al., 2014; Raymond-Bouchard et al., 2018). These data suggest that multiple molecular strategies have evolved to cope with common environmental stressors in polar ecosystems.

The low concentrations of micronutrients, specifically iron, compared with macronutrients in the Southern Ocean results in a High Nutrient Low Chlorophyll region (HNLC) which limits phytoplankton growth (Martin et al., 1991; Pitchford and Brindley, 1999; Venables and Moore, 2010; Hassler et al., 2012). Thus, iron limitation can impose cellular stress. For example, iron limitation compounds the intracellular ROS stress due to a reduction in the capacity of the electron transport chain (Allen et al., 2008). Genomics, transcriptomics, and proteomics studies have discovered unique proteins which are responsive to micronutrient fluxes to mitigate this stress such as through FLDA2b in *F. kerguelensis* (Allen et al., 2008; Marchetti et al., 2009; Lommer et al., 2012; Bender et al., 2014; Marchetti, 2019; Moreno et al., 2020). Southern Ocean diatoms exposed to varying light intensities, and different iron concentrations, were found to have an increased number of genes associated with iron uptake compared to temperate diatoms (Mock et al., 2017; Moreno et al., 2018). Iron concentrating proteins in polar diatoms, such as iron starvation-induced protein 2a (ISIP2a) identified in *F. kerguelensis*, are significantly overexpressed in low iron conditions to increase uptake of iron from the environment (Marchetti et al., 2009; Moreno et al., 2020). In addition to increased affinity for iron, the *F. cylindrus* genome is enriched for copper-binding domains, outnumbering iron-binding domains (Mock et al., 2017). Most are of the plastocyanin/azurin-like family, possibly reducing the requirement for iron in cellular processes (Peers and Price, 2006; Mock et al., 2017). *F. kerguelensis* contains several plastocyanin isoforms which are significantly upregulated in response to low iron, specifically *PCYN-2b* (Moreno et al., 2020). The *F. cylindrus* genome also revealed an elevated number of zinc-binding domain-containing genes, with more than six times the number of homologous clusters compared to *P. tricornutum* and *T. pseudonana*; specifically, MYND Zinc Fingers. MYND domains in *F. cylindrus* are associated with a variety of accompanying domains with diverse functions, many of which are unknown (Laity et al., 2001; Mock et al., 2017). The evolutionary expansion of this family suggests their importance potentially in terms of regulating the activity of gene expression and/or the activity of enzymes (Mock et al., 2017). Their expansion may have been facilitated by the relatively high zinc concentrations in the Sothern Ocean (Croot et al., 2011; Mock et al., 2017). Most of the expansion in this family is predicted to have originated around 30 million years ago, which therefore coincides with the opening of the Drake passage (Mock et al., 2017). Thus, the coincidence between the evolutionary expansion of zinc-binding protein genes and the cooling of the Southern Ocean begs the question about the role of zinc in the adaptation of diatoms to environmental conditions in surface polar oceans.

The studies discussed so far in this review have given insight into the viability and usefulness of polar model organisms to reveal fundamental processes underpinning adaptation and evolution, however, relying on model organisms limits our ability to understand how biodiversity changes due to environmental change. For instance, the role of microbial interactions is neither well studied nor represented by using a monoculture of an individual model species (Wolf et al., 2019) because intra- and interspecific competition is based on complex dynamic populations and their communities shaping the evolution and adaptation of all microbes involved. For example, in the study by Wolf et al. (2019), different strains of the species *Thalassiosira hyaline* were grown in monoculture under predicted future climate conditions and then mixed with other strains to see if their physiology changed when cultivated altogether. Under elevated temperatures, the fastest-growing strain outcompeted the others when grown in mixed culture. Conversely, under ambient temperatures, its growth rate resembled that of the slowest growing strain (Wolf et al., 2019). This demonstrates that monoculture studies may not be representative of how individual species behave as part of complex communities in nature. Thus, understanding intraspecific variation between strains of the same species is important because differences in traits might shape the structure and function of food webs and therefore biogeochemical cycling of elements (Vance et al., 2019). Hence, differences in the co-occurrence of strains and species in complex microbial communities are important for considering how environmental conditions have shaped these communities (e.g., Martin et al., 2021). To address this question, meta-omics approaches have proven successful in revealing the intricacies of complex microbial communities and their activity.

## Polar Meta-Omics

### Metagenomes and Their Assembled Genomes

Metagenomics, metatranscriptomics, and metaproteomics are emerging as important tools for understanding the molecular basis of adaptation in polar microbes by allowing us to study organisms in their ecosystem and to capture their interactions in the natural environment (McCain et al., 2021). Metagenomics has been used to establish which diatom species are present in certain locations, providing a baseline for future monitoring systems. For example, the Tara Oceans project investigated the global distribution of diatoms and discovered unexpected species in polar oceans (Malviya et al., 2016; Carradec et al., 2018; Obiol et al., 2020). Similarly unexpected was that polar oceans are a particular hotspot of viral diversity with high levels of novel genes (Gregory et al., 2019). Thus, metagenomics helps to reveal the microbial biodiversity in polar oceans and how different it is when compared to metagenomes from non-polar oceans. Metagenomics has also been applied to provide insights into how polar environmental gradients (e.g., sea-ice water interface) influence microbial biodiversity. For instance, a metagenomics survey of microbial communities in the Canadian Arctic found that in sea-ice there was a higher concentration of algal genes and chlorophyll-a compared to seawater underneath, where prokaryotic genes were more dominant. Metagenomic data showed that this was largely due to diatoms. Differences in operational taxonomic units (OTUs), a group of closely related individuals, between sea-ice and sea-water samples indicated that sea-ice dwellers have different strategies for adaptation than seawater-based microbes. Increased variability was found in the sea-ice communities, perhaps indicative of the adaptability of polar diatoms as a dominant and diverse group of microbes in the sea-ice habitat (Yergeau et al., 2017).

Sequencing of individual species such as *F. cylindru*s, *P. glacialis, and Chlamydomonas sp.* UW0241 (Figure 2), has advanced our fundamental understanding of polar algae (Bayer-Giraldi et al., 2010; Mock et al., 2017; Stephens et al., 2020; Zhang et al., 2021). However, their sequencing requires resources and takes time. Yet, they serve as a reference not only for fundamental research but also for analyzing phytoplankton-enriched metagenomes. The latter together with reference genomes therefore will allow for the assembly of novel algal genomes isolated from natural communities including their associated microbiomes. Insights from this work will help us to understand the similarities and differences between genomes from natural communities, rather than relying on model organisms which risks not representatively capturing the diversity of adaptive mechanisms underlying their complex intertwined co-evolution. For instance, targeted metagenomics has been used to establish the evolutionary history of picoprymnesiophyte populations in the North Atlantic, which make up approximately 25% of global pico-plankton biomass. Their dominance was found to be due to high gene density, and genes that are likely to be involved in defense and nutrient uptake (Cuvelier et al., 2010).

Metagenome-assembled genomes (MAGs) of prokaryotes have been produced using large quantities of metagenomic data from both the Arctic and Antarctic (Cao et al., 2020; Royo-Llonch et al., 2021). Increased availability of high-quality MAGs without the requirement for individual culturing and sequencing will allow researchers to study a broader, more representative range of polar microbiomes and compare between species to understand mechanisms for polar survival (Cao et al., 2020; Duncan et al., 2020). MAGs have been used to study polar bacterial and archaeal populations from the Tara Oceans dataset, providing the first compendium of Arctic prokaryotic MAGs including differences in gene enrichment between Arctic and Antarctic populations (Cao et al., 2020; Royo-Llonch et al., 2021).

Although most MAGs from polar oceans are still from prokaryotes, one of the first MAG-based datasets from natural Arctic and Atlantic microbial communities was used to reveal inter-kingdom species associations including microbial eukaryotes (Duncan et al., 2020). Besides the generation of first eukaryotic MAGs from a polar ocean (e.g., diatoms, prasinophytes), this dataset was used to identify metabolism enriched in MAG-based species associations. By identifying which protein families are enriched in selected species, comparisons of associations to a background set of phylogenetically related species known not to have associations shed first insights into shared metabolism, potentially underpinning their biotic interactions. By applying this approach, Duncan et al. (2020) found positive and negative associations between algal and bacterial MAGs including some that were only found in the Arctic (e.g., Micromonas MAG associated with a Gammaproteobacteria MAG). Combined with cell isolations from the same samples, these MAG-based species associations can be empirically tested if the microbial partners can be co-cultivated under laboratory conditions.

### Metatranscriptomes and Metaproteomes

Investigation of the genes expressed across global oceans was carried out as part of the Tara Oceans project (Bork et al., 2015) and the Sea of Change project (Martin et al., 2021). The former has been used to create an ocean atlas of eukaryotic genes from temperate and tropical regions whereas the latter has been used to produce the first pole-to-pole catalog of expressed genes from microalgae and other microeukaryotes. Most of the expressed genes in the Tara Oceans Atlas were novel, indicating that eukaryotic ocean life is under-studied and therefore incompletely understood. Additionally, genes specific to the Southern Ocean were different to those found elsewhere, potentially indicating that adaptation to polar conditions results in a very different gene set to non-polar species (Carradec et al., 2018). The latter insights were corroborated and extended by the Sea of Change project, which revealed that global algal microbiomes can be largely separated into two main groups: polar and non-polar species associations. The same demarcation was found for expressed genes of the algal partners considering their biogeographic distribution from pole to pole based on a combination of sequence co-occurrence analysis and the geographical location of breakpoints in their beta diversity (Difference in species composition between neighboring assemblages) (Martin et al., 2021). Thus, it appears that there are ecosystem boundaries in sub-polar regions of the upper ocean in both hemispheres separating polar from non-polar algal microbiomes.

Although metaproteomics with natural polar microbial communities is still in its infancy, the first study on coastal Antarctic phytoplankton communities has been undertaken. This study by McCain et al. (2021) used proteome-level traits to identify ecological strategies for different taxa. For instance, haptophytes appear to have a lower regulatory cost than diatoms, which may explain the observed haptophyte-to-diatom bloom progression in the Ross Sea. As protein synthesis is the main energy sink in cells, the quantity of cellular proteins measured as part of complex metaproteomes relates to costs involved in their synthesis. Hence, the nature and quantity of specific proteins identified potentially provides insights into the costs of traits and trade-offs. Challenges arise, however, with incomplete taxon-specific proteomes as part of complex metaproteomes especially if reference data are missing.

### Omics-Based Monitoring and Modeling to Improve Prediction of Species’ Responses to Polar Warming

Genetic monitoring using metagenomics and metatranscriptomics of mixed communities to oversere changes in populations over time can be used to study adaptations to changing environmental conditions, showing the rate of evolution over time and how adaptations are occurring (Hansen et al., 2012; Pearson et al., 2015). However, sequence-based monitoring of microbial communities and their populations needs to be carefully designed to identify the most appropriate sites for capturing changes in the biogeography of species and their populations including alterations of gene flow and climate-driven range-shift expansions in their distribution. Reoccurring surveys with sufficient spatial sampling in a given geographic area or multiple monitoring sites with frequent sampling will be needed for a rigorous assessment of how warming in polar oceans influences microbial populations and the biogeochemical cycles they drive. Furthermore, monitoring needs to cover complete seasonal cycles to allow us to tease apart long-term ecosystem change from seasonal effects, which might be challenging as the latter are much more pronounced at high latitudes. Although many different geographical sites have been used for sampling and monitoring polar microbial communities, two stand out: The Fram Observatory in the Arctic and long-term observation of plankton at the Western Antarctic Peninsula (Lin et al., 2021; Wietz et al., 2021). The Fram observatory is based on autonomous samplers, which can sample at discrete time intervals to cover a complete seasonal cycle. Preliminary results covering 12 months including the dark winter period have revealed that the strong seasonal dynamics including the variable sea-ice extent are the main drivers for the succession in changes of microbial biodiversity based on 16 and 18S rDNA data and subsequent analysis (e.g., alpha diversity). Furthermore, the persistent sea-ice cover appears to reduce the seasonality effect on changes in microbial biodiversity (Wietz et al., 2021). As the autonomous samplers can be redeployed, they provide a platform for long-term monitoring of microbial communities in the Arctic Ocean. A similar monitoring system is not yet, to our knowledge, available for the Southern Ocean, although there is a ship-based multi-year monitoring programme at the Western Antarctic Peninsula (Lin et al., 2021) based on similar methods (e.g., 16/18S rDNA). Modeling has also been shown that sea ice plays a significant role in structuring microbial communities and their productivity (Lin et al., 2021). However, increasing temperatures due to warming in this region are likely to result in declining microbial diversity (Tonelli et al., 2021).

Both of these monitoring programs have provided novel and highly needed insights into how the environmental conditions of the changing polar oceans shape their microbial inventory. To further understand how biotic and abiotic forces determine the abundance and distribution of microbial taxa in a polar ocean, future studies need to provide quantitative insights into how microbial traits (e.g., freeze-thaw resistance) underpin species interactions and how they are associated with habitat characteristics (e.g., sea-ice thickness, oceanic fronts, temperature gradients, snow-cover thickness). These biotic and abiotic forces likely encompass speciation, dispersal, and biotic interactions in a complex and highly dynamic polar environment.

**FIGURE 3:**
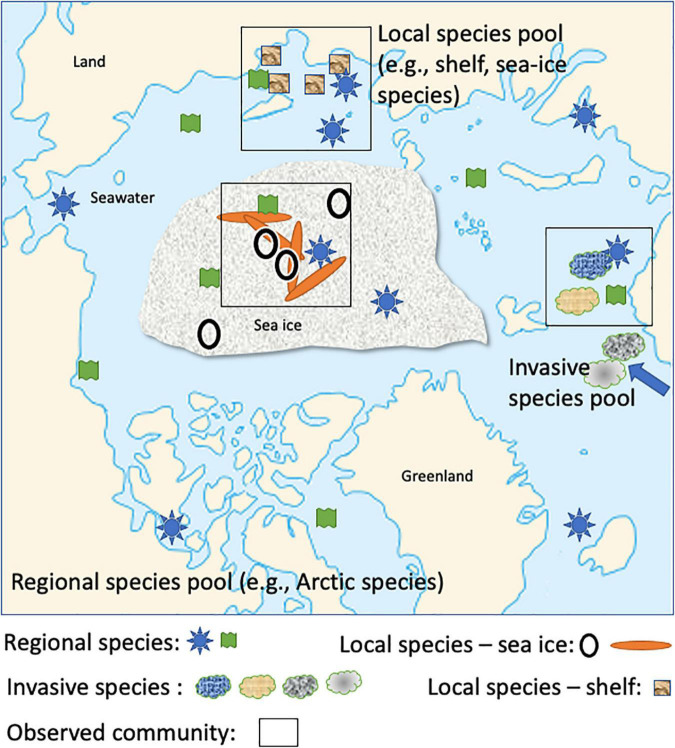
Conceptual diagram of the assembly process influencing microbial communities at different spatiotemporal scales in the Arctic Ocean (e.g., regional, local). Geometric forms are arbitrary, but their composition represents how different microbial taxa (e.g., a specific geometric form such as a circle representing a taxon) contribute to complex microbiomes as the outcome of assembly processes at different spatiotemporal scales (Adapted from Ovaskainen et al., 2017). Drivers for the assembly process are likely a combination of abiotic and biotic conditions. For instance, the ephemeral nature of the sea-ice habitat in combination with strong gradients of temperature and salinity in brine channels can be assumed to significantly contribute to the assembly process of sea-ice microbiomes.

The responses of microbial taxa to different habitat characteristics (environmental filters) and biotic interactions (biotic filters) vary depending on taxa-specific traits including their ability to acclimate, adapt, and their level of competitiveness (Ovaskainen et al., 2017). Thus, these traits will determine which taxa colonize habitats and dominate in seasonal successions (phenology) and therefore contribute to the community assembly process. To address this fundamental question, it is instrumental to apply explanatory models which can integrate data generated for quantitative insights into how microbial communities including their networks relate to environmental conditions and larger-scale ecosystem processes (e.g., seasonality of microbial diversity linked with habitat transformations such as freezing and melting of sea ice) (Ovaskainen et al., 2017; Tikhonov et al., 2020). One approach to tackle this challenge is hierarchical joint species/taxa distribution modeling (HJSDM) (Tikhonov et al., 2020). The heart of this approach is integrating species co-occurrence in co-variation with environmental conditions, and phylogeny of species and traits. Data required for this approach can be extracted from metagenomes, metatranscriptomes, and metaproteomes. For instance, MAGs will provide a genomic catalog (e.g., Cao et al., 2020; Royo-Llonch et al., 2021) of co-occurring microbial taxa in a phylogenetic but also spatio-temporal context if based on long-term sampling. Their genes, transcripts, and proteins provide trait information for revealing co-variation with environmental conditions. If sampling covers larger geographic regions, as done for the West Antarctic Peninsula, or the central Arctic Ocean as part of MOSAiC (Multidisciplinary drifting Observatory for the Study of Arctic Climate) (Wake, 2019), we will be able to delineate processes influencing the biodiversity of microbial communities at different spatio-temporal scales (Figure 3). Generally, a spatio-temporal context is required for separating drivers of seasonal differences vs climate-driven differences in microbial community composition and therefore to make robust predictions as to how warming impacts polar ecosystems.

## Discussion

The Earth’s climate has been changing across the globe due to anthropogenic influences since the industrial revolution. There is no doubt of the importance of polar environments to regulate the global climate, with the Southern Ocean alone responsible for the uptake of around 40% of all anthropogenic CO_2_ from the atmosphere (Khatiwala et al., 2009; Takahashi et al., 2009; DeVries, 2014; Long et al., 2021). As previously mentioned, polar ecosystems are experiencing more rapid changes, specifically warming, than any other biome on Earth. This alarming shift has been met with increased research over recent decades to understand the physical properties of these environments and the effect they have on food webs, with a focus on marine microbiomes, and diatoms in particular as one of the most important primary producers in polar oceans. Genome-enabled approaches have provided a step-change in our understanding of the adaptability of polar diatoms and allowed us to understand how they reacted to past climate events. However, there is still only one publicly available polar diatom genome, *F. cylindrus*, and with > 350 species of phytoplankton found in the Southern Ocean alone, using one genome to study such a diverse community is not viable. Hence, these studies need to expand as exemplified by the 100 Diatom Genomes Project^1^ and the MOSAiC sequencing project at the United States Department of Energy Joint Genome Institute^2^. If monitoring programs build on these initiatives and adopt high-throughput omics approaches, we can generate the foundation for cataloging polar microbiomes through space and time. This information will help to integrate species association networks with traits to provide quantitative insights into how traits of species and their co-occurrence networks are associated with habitat characteristics. Based on this integrated approach, we potentially will be able to predict the future of microbial taxa and their diverse populations with greater confidence in warming polar oceans.

## Author Contributions

RG, EL, and TM wrote the article and equally contributed to the design of the figures. All authors contributed to the article and approved the submitted version.

## Conflict of Interest

The authors declare that the research was conducted in the absence of any commercial or financial relationships that could be construed as a potential conflict of interest.

## Publisher’s Note

All claims expressed in this article are solely those of the authors and do not necessarily represent those of their affiliated organizations, or those of the publisher, the editors and the reviewers. Any product that may be evaluated in this article, or claim that may be made by its manufacturer, is not guaranteed or endorsed by the publisher.

## Footnotes

1
https://jgi.doe.gov/csp-2021-100-diatom-genomes/

2
https://jgi.doe.gov/csp-2020-arctic-ice-drift-experiment-mosaic/
